# A Heterometallic Three-Dimensional Metal−Organic Framework Bearing an Unprecedented One-Dimensional Branched-Chain Secondary Building Unit

**DOI:** 10.3390/molecules25092190

**Published:** 2020-05-07

**Authors:** Jing Chen, Meng-Yao Chao, Yan Liu, Bo-Wei Xu, Wen-Hua Zhang, David J. Young

**Affiliations:** 1 College of Chemistry, Chemical Engineering and Materials Science, Soochow University, Suzhou 215123, China; chenjing120905003@163.com (J.C.); chaomy@mail.sustech.edu.cn (M.-Y.C.); liuyanllllll@163.com (Y.L.); huhulalaxu@163.com (B.-W.X.); 2 College of Engineering, Information Technology & Environment, Charles Darwin University, Darwin, Northern Territory 0909, Australia; david.young@cdu.edu.au

**Keywords:** metal-organic framework, heterometallic cluster, heptanuclear cluster, calcium cluster, cadmium cluster, crystal structure

## Abstract

A heterometallic metal−organic framework (MOF) of [Cd_6_Ca_4_(BTB)_6_(HCOO)_2_(DEF)_2_(H_2_O)_12_]∙DEF∙*x*Sol (**1**, H_3_BTB = benzene-1,3,5-tribenzoic acid; DEF = *N*,*N*′-diethylformamide; *x*Sol. = undefined solvates within the pore) was prepared by solvothermal reaction of Cd(NO_3_)_2_·4H_2_O, CaO and H_3_BTB in a mixed solvent of DEF/H_2_O/HNO_3_. The compatibility of these two divalent cations from different blocks of the periodic table results in a solid-state structure consisting of an unusual combination of a discrete V-shaped heptanuclear cluster of [Cd_2_Ca]_2_Ca′ and an infinite one-dimensional (1D) chain of [Cd_2_CaCa′]_n_ that are orthogonally linked via a corner-shared Ca^2+^ ion (denoted as Ca′), giving rise to an unprecedented branched-chain secondary building unit (SBU). These SBUs propagate via tridentate BTB to yield a three-dimensional (3D) structure featuring a corner-truncated *P*4_1_ helix in MOF **1**. This outcome highlights the unique topologies possible via the combination of carefully chosen s- and d-block metal ions with polydentate ligands.

## 1. Introduction

Heterometallic metal−organic frameworks (MOFs) merge the traits of two or more different metal ions in one ordered array with synergistic advantages that can be applied to catalysis [1,2,3,4,5,6,7,8], gas adsorption [9,10,11,12,13], bioimaging [14,15,16], and other applications [17,18,19,20,21]. The heterometal can be introduced by the direct one-pot assembly of mixed metal sources and ligands [22,23,24,25,26,27,28,29], or from preformed secondary building units (SBUs) [17,18,30,31,32,33,34,35,36]. Post-synthetic metal coordination to the functionalized ligands [37,38,39,40,41,42,43,44,45,46] and transmetalation/incorporation of ions into the SBUs have also been reported [3,24,42,47,48,49,50].

The presence of a second metal ion in the one-pot assembly may play a structure-directing role in obtaining MOF topologies inaccessible from the individual metal sources [30,31,32,33,34,51]. By comparison, the post-synthetic method is better for obtaining MOFs with predictable topology, and the targeted metal ion can diffuse into the pores to trigger the cation incorporation/exchange without disrupting the overall framework connectivity [49,52,53,54,55,56,57,58,59]. The one-pot reaction faces the risk of forming separate MOFs from dependent metal ions, whereas the post-synthesis method has the challenge of incomplete conversion, requiring repeated experiments to obtain a desirable conversion efficiency.

The one-pot assembly of heterometallic MOFs is facilitated by metal ions of similar ionic radius and charge. Mixtures of Zn^2+^ (0.070 ± 0.007 nm) and Mg^2+^ (0.070 ± 0.004 nm) or Cd^2+^ (0.0910 ± 0.003 nm) and Ca^2+^ (0.103 ± 0.005 nm) may have promising advantages in this respect [60]. However, there are only limited reports of heterometallic MOFs of these elements, presumably because of the relatively few exploitable properties of Mg^2+^ and Ca^2+^.

Incorporating s-block metal ions, such as Li^+^, Na^+^, Mg^2+^, and Ca^2+^, into MOF assemblies may impart several advantages. For example, the flexible coordination geometries of these ions and the ionic forces between the metal ions and ligands (relative to dative bonds between a typical d-block metal ion and the ligand) may permit novel framework topologies. Some of these s-block metal ions (e.g., Li^+^) have additional advantages of being inexpensive and relatively non-toxic [12,50,61].

In this work, we report a three-dimensional (3D) MOF of [Cd_6_Ca_4_(BTB)_6_(HCOO)_2_(DEF)_2_(H_2_O)_12_]∙DEF∙*x*Sol. (**1**, H_3_BTB = benzene-1,3,5-tribenzoic acid; DEF = *N*,*N*′-diethylformamide; *x*Sol. = undefined solvates within the pore) sustained by an unprecedented one-dimensional (1D) branched SBU, which, in turn, consists of a discrete V-shaped heptanuclear cluster of [Cd_2_Ca]_2_Ca′ and an infinite 1D chain of [Cd_2_CaCa′]_n_ linked via a corner-shared Ca^2+^ ion (denoted as Ca′). Such a complicated MOF generated via the mixed use of a Cd^2+^ and Ca^2+^ features the first case of a framework structure containing both Ca^2+^ and BTB ligands. In addition, the structure of MOF **1** is dramatically different from, and in fact much more delicate than, all the literature examples that are exclusively from the assembly of Cd^2+^ with BTB ligands [62,63,64,65].

## 2. Results and Discussion

### 2.1. Synthesis and Material Characterization of MOF **1**

The motivation for introducing Ca^2+^ into a Cd^2+^/H_3_BTB assembly originated from our quest to incorporate Ca^2+^ into the linear Cd_3_-based two-dimensional (2D) MOF of [Cd_3_(BTB)_2_(DEF)_4_]·2(DEF)_0.5_ [62,63], as we and others have demonstrated that the heterometallic combinations of Cd/Zn, Cd/Co, and Zn/Co are readily tolerated in linear trimetallic cluster SBUs [26,66]. Our previous trial of the solvothermal reaction of Cd(NO_3_)_2_∙4H_2_O and H_3_BTB in a DEF/H_2_O/HNO_3_ mixed solvent with CaSO_4_ yielded a 3D anionic MOF of [Et_2_NH_2_]_2_[Cd_5_(BTB)_4_(DEF)_2_]∙4.75DEF featuring a zigzag-shaped Cd_5_ cluster SBU [65]. The [Et_2_NH_2_]^+^ cations likely arose from the acid-catalyzed hydrolysis of DEF [67,68,69,70], and subsequently served as a template for the framework formation. In the present work, we employed CaO as a replacement of CaSO_4_ to consume part of the acid, and the new neutral 3D MOF **1** was thus obtained in a high yield of 87%. It is interesting to note that DEF also decomposed in the present case, with the anionic part of HCOO^−^ acting as a bridging ligand for structure propagation.

**Figure 1:**
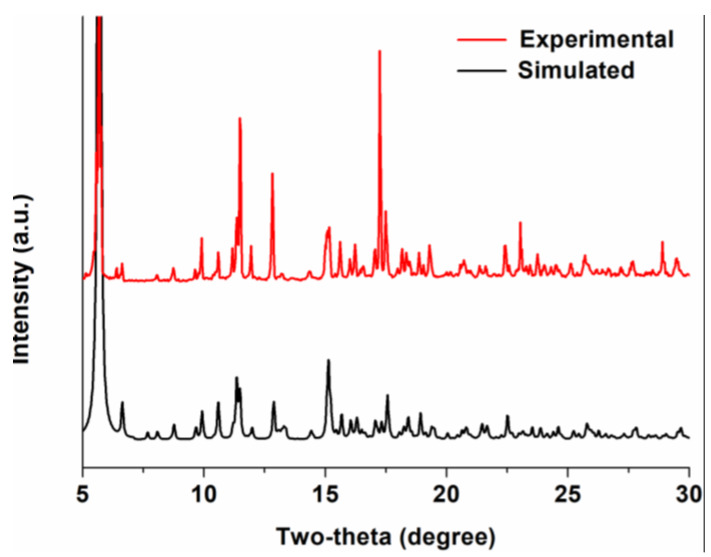
The powder X-ray diffraction patterns of MOF **1**, showing a good consistency between the experimental (red) and simulated (black) patterns and thus a high phase purity of the bulk material. The slight inconsistency in the intensity of some peaks might have been due to the different orientation of the crystallites in the sample, as the metal–organic framework (MOF) is non-merohedrally twinned.

MOF **1** contains Ca^2+^ as a key component, is stable under aerobic conditions, and is stable and insoluble in MeOH, EtOH, DMF, DEF, CH_2_Cl_2_, CHCl_3_, and MeCN. The powder X-ray diffraction (PXRD) pattern of the bulk crystalline sample of MOF **1** agreed well with that simulated from the single-crystal diffraction data, indicating its bulk phase purity (Figure 1). The thermogravimetric analysis (TGA) of MOF **1** indicated that the framework is stable up until ca. 100 °C (Supplementary Figure S1), followed by a continuous weight loss up to ca. 650 °C. At this stage, we were not able to provide a clear compositional analysis based on the TGA result due to the large pore cavity of MOF **1** (as will be discussed below). It is also unfortunate that there was no obvious plateau found upon initial solvate loss. The thermally unstable nature of MOF **1** prevented us from studying its surface area via gas adsorption, and our preliminary experiments suggested that MOF **1** exhibits no adsorption of N_2_ at 77 K (Supplementary Figure S2), presumably due to the collapse of the 3D framework. The Fourier-transform infrared spectrum (FT-IR) of MOF **1** contained absorptions at 2975 cm^−1^, 2930 cm^−1^, and 1652 cm^−1^ corresponding to the–CH_3_, –CH_2_–, and –C=O bonds of te DEF solvate [71,72]. In addition, peaks at 1610 cm^−1^, 1536 cm^−1^, and 1397 cm^−1^ were assignable as the asymmetrical and symmetrical stretching vibrations of the carboxylate [66,73].

### 2.2. Crystal Structure Analysis of MOF **1**

The presence of Cd^2+^ and Ca^2+^ and their exact atomic sites in MOF **1** could be unambiguously inferred from the X-ray crystallographic studies. This benefited from the large discrepancy of the atomic mass between Cd^2+^ (112.441) and Ca^2+^ (40.078), as any incorrect assignment of the atomic sites will lead to strikingly unrealistic thermal ellipsoids and other related issues during the structure refinement. Such MOF assemblies from a mixture of Cd^2+^ and Ca^2+^ sources thus have an advantage for structure determination by X-ray crystallography, in stark contrast to those heterometallic MOFs bearing mixed metals with similar atomic mass, such as Zn/Co and Mn/Co [23,66].

**Figure 2:**
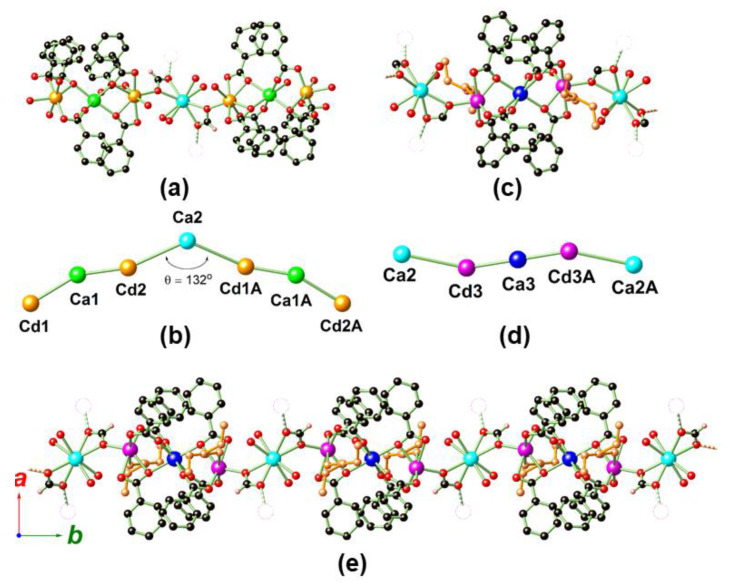
The structures of the discrete V-shaped heptanuclear cluster (**a**,**b**) and 1D rod-shaped (**c**–**e**) subunits in MOF **1**, showing the full coordination geometry around each metal (**a**,**c**,**e**), and the framework skeleton represented by only metals (**b**,**d**). In (**a**), dashed bonds with blank spheres indicate that the heptanuclear cluster is orthogonally associated with the 1D rod-shaped chain via a pair of HCOO with *μ*_3_-*η*^1^:*η*^1^:*η*^2^ coordination, and vice versa (**c**,**e**). All disordered components, dissociated DEF solvate, and hydrogen atoms (except those on HCOO) are omitted for clarity. Color legend: C: black, H: light pink, O: red, Cd1/Cd2: orange, Cd3: magenta, Ca1: green, Ca2: cyan, Ca3: blue. The coordinated DEF solvates in (**c** and **e**) are further distinguished with orange color for clarity. Symmetrical code to generate equivalent atoms in (**b**): −*x*, −*y* + 1/2, *z*, and (**d**): −*x*, −*y* + 1, −*z* + 2.

MOF **1** crystallizes in the tetragonal crystal system space group *I*4_1_/*a* (Table 1), and its asymmetrical unit contains three Cd^2+^, three Ca^2+^, three BTB and one HCOO bridging ligands, together with one coordinated DEF (to Cd3) and six coordinated H_2_O solvates (two to Cd1, one to Cd2, one to Ca1, and two to Ca2). There is also one DEF solvate with half occupancy residing within the cavity of MOF **1**. As shown in Figure 2a,b, the discrete V-shaped heptanuclear [Cd_2_Ca]_2_Ca′ cluster contains a pair of trinuclear [Cd_2_Ca] (Cd1, Cd2 and Ca1) clusters symmetrically related by one additional Ca′ center (Ca2) ([Cd_2_Ca]:Ca′ = 2:1, or, alternativelym Cd^2+^:Ca^2+^ = 4:3). Within the [Cd_2_Ca] cluster, Cd1 (6-coordinate) is coordinated by three distinctive carboxylates, i.e., one monodentate (*η*^1^), one bridging (*μ*_2_-*η*^1^:*η*^1^), and one chelating-bridging (*μ*_2_-*η*^1^:*η*^2^), in addition to two H_2_O solvates that terminate the propagation of the cluster. Cd2 (7-coordinate) is associated with one bridging (*μ*_2_-*η*^1^:*η*^1^) and a pair of chelating-bridging (*μ*_2_-*η*^1^:*η*^2^) carboxylates, one chelating-bridging HCOO (*μ*_3_-*η*^1^:*η*^1^:*η*^2^), and one additional coordinated H_2_O. In addition, the central Ca1 (6-coordinate) is associated with two bridging (*μ*_2_-*η*^1^:*η*^1^) and three chelating-bridging (*μ*_2_-*η*^1^:*η*^2^) carboxylates, in addition to one terminally coordinated H_2_O. On the other hand, the central Ca2 (Ca′, 8-connected) is associated with a pair of chelating-bridging HCOO (*μ*_3_-*η*^1^:*η*^1^:*η*^2^) and four terminal H_2_O solvates. The [Cd_2_Ca]-Ca′-[Cd_2_Ca] angle (θ) is roughly 132° (Figure 2b).

The 1D rod-shaped [Cd_2_CaCa′]_n_ chain also contains a [Cd_2_Ca] cluster unit and one Ca2 (Ca′) center which are alternatively arranged ([Cd_2_Ca]:Ca′ = 1:1, or, alternatively, Cd^2+^:Ca^2+^ = 1:1) (Figure 2c–e). Within the chain, the Ca2 (Ca′) ion is corner-shared with the V-shaped heptanuclear [Cd_2_Ca]_2_Ca′ cluster (Figure 2a,b) as discussed. The [Cd_2_Ca] cluster unit herein comprises a pair of Cd centers (Cd3) symmetrically related via the central Ca3, in which Cd3 (6-coordinate) is associated by a pair of bridging (*μ*_2_-*η*^1^:*η*^1^) and one chelating-bridging (*μ*_2_-*η*^1^:*η*^2^) carboxylates, one chelating-bridging HCOO (*μ*_3_-*η*^1^:*η*^1^:*η*^2^), and a terminally coordinated DEF solvate. Meanwhile, the central Ca3 (6-coordinate) is bonded by six carboxylates, of which four are in bridging (*μ*_2_-*η*^1^:*η*^1^) and two in chelating-bridging (*μ*_2_-*η*^1^:*η*^2^) coordination fashions.

**Figure 3:**
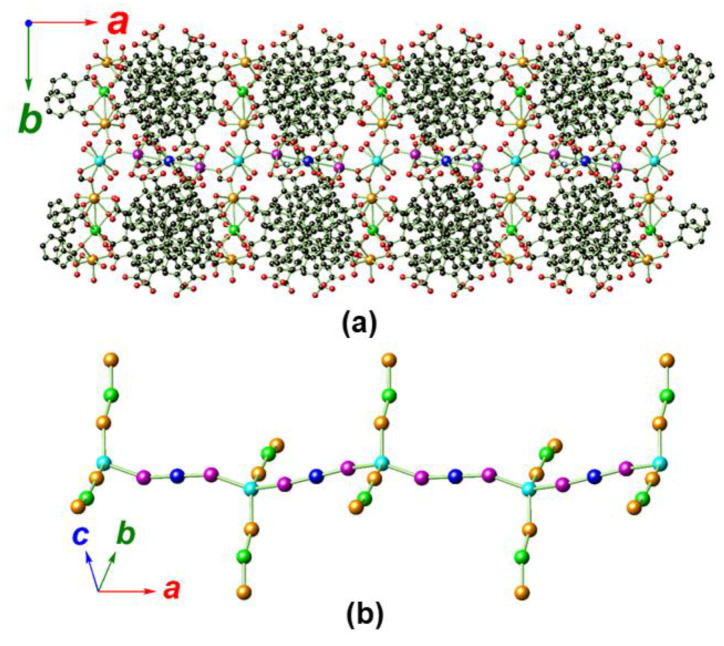
The 1D branched-chain SBU of MOF **1** propagated along the crystallographic *a* direction with associated BTB ligands (**a**) and metal skeleton (**b**). All disordered components, dissociated DEF solvate, and hydrogen atoms are omitted for clarity. Color legend: C: black, O: red, Cd1/Cd2: orange, Cd3: magenta, Ca1: green, Ca2: cyan, Ca3: blue.

**Figure 4:**
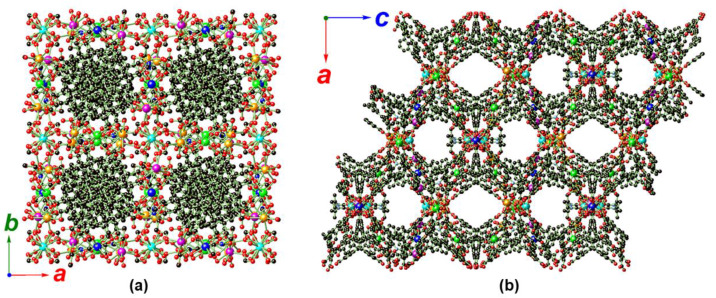
Crystal packing diagrams of MOF **1** looking along the crystallographic *c* direction, showing the densely packed BTB ligands (**a**), and along the crystallographic *b* direction, showing the channels with rhombohedral apertures (**b**). All disordered components, dissociated DEF solvate, and hydrogen atoms are omitted for clarity. Color legend: C: black, N: light blue, O: red, Cd1/Cd2: orange, Cd3: magenta, Ca1: green, Ca2: cyan, Ca3: blue.

The intersection of the discrete V-shaped heptanuclear [Cd_2_Ca]_2_Ca′ subunits and the rod-shaped [Cd_2_CaCa′]_n_ chain through Ca′ (Ca2) gives rise to a 1D branched SBU (Figure 3a,b). Such an unprecedentedly broad SBU features isolated voids which are filled with the bulky BTB ligands. It may be that these BTB ligands serve as the template for the arrangement of this unique SBU. These sizable SBUs are further associated with BTB ligands to give a 3D framework structure featuring densely packed BTB ligands when looking along the crystallographic *c* direction (Figure 4a). When looking along the crystallographic *a* or *b* direction, 1D corner-sharing channels with rhombohedral apertures are observed (Figure 4b). A Platon void calculation indicates a total solvent-accessible volume (including the free DEF) for each cell unit amounting to 22,138.0 Å^3^ or 43.3% of the total cell volume (51,134.0 Å^3^) [74].

**Figure 5:**
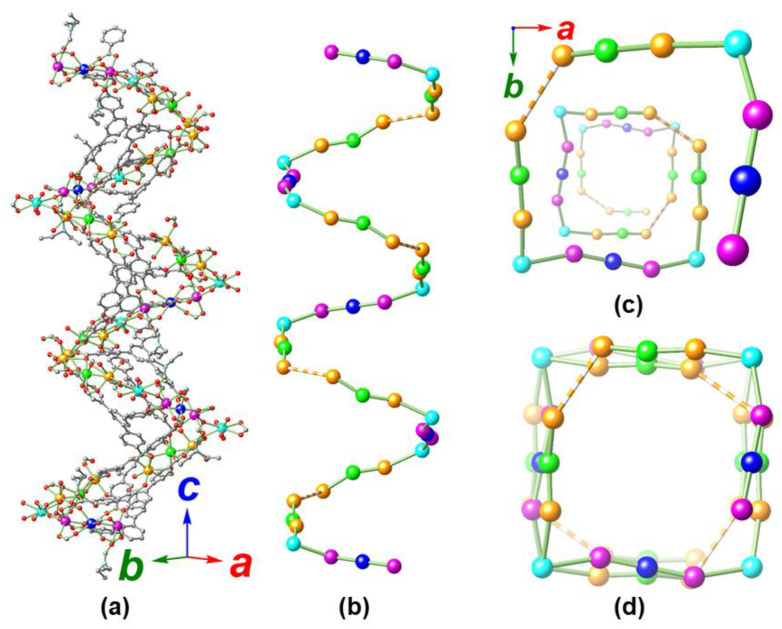
Structures of MOF **1** showing a helical chain propagating along the *c* direction assisted by the BTB ligands (**a**), skeleton structure with only metals (**b**), and the perspective (**c**) and parallel (**d**) views of the helical chain along the *c* direction. Dashed bonds are used in (**b**–**d**) to indicate imaginary connections between Cd1 and its equivalents to facilitate understanding of the structure. All disordered components, dissociated DEF solvate, and hydrogen atoms are omitted for clarity. Color legend: C: gray, N: light blue, O: red, Cd1/Cd2: orange, Cd3: magenta, Ca1: green, Ca2: cyan, Ca3: blue. The BTB ligand skeletons and coordinated DEF in (**a**) are further distinguished by gray for clarity.

It is interesting to note that the two types of [Cd_2_Ca] cluster units (Cd1-Ca1-Cd2 denoted as type A node, and Cd3-Ca3-Cd3 denoted as type B node; ratio type A:type B = 2:1) propagate in the *c* direction in a helical sequence around the 4_1_ axis (Figure 5). In such a helical chain, the Ca′ (Ca2, denoted as type C node) is critical and serves as the corner mediating the turn between type A and type B nodes. However, an additional corner to mediate the turn between two sequential type A nodes is missing (we herein use node □ to represent such an imaginary corner). These four types of node (A, B, C, and □) are linked in a —[∙∙∙A□ACBC A□ACBC∙∙∙]_n_— fashion with an extremely long helical pitch of 54.251(4) Å (four A□ACBC sequences, equal to the crystallographic *c* distance) containing 44 metal ions (24 Cd and 20 Ca, respectively, Figure 5b,c).

**Figure 6:**
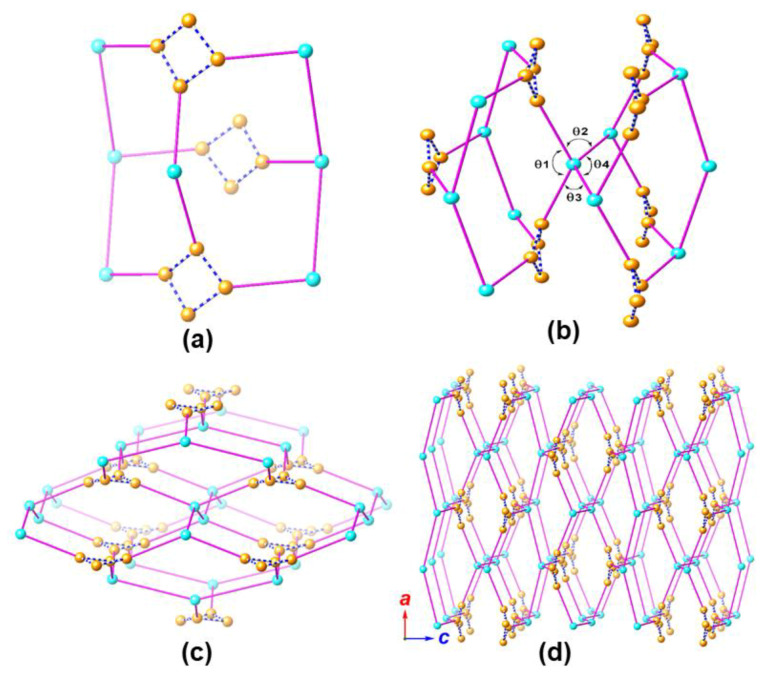
Topological presentations of MOF **1**, with the [Cd_2_Ca] subunits (Cd1-Ca1-Cd2) presented as orange spheres, and Ca2 ions that mediate the association of the V-shaped heptanuclear cluster and 1D rod-shaped chain presented as cyan spheres. The [Cd_2_Ca] subunits (Cd3-Ca3-Cd3) within the 1D rod-shaped subunits have no topological contribution and are thus omitted. A set of four [Cd_2_Ca] subunits (Cd1-Ca1-Cd2) are spatially clustered and they are grouped using dashed lines to facilitate topological understanding. These subunits function either as the edge ([Cd_2_Ca] cluster within the 1D rod-shaped chain), or the vertices (Ca2 or four [Cd_2_Ca] clusters of the heptanuclear subunits that collectively serve as one vertex) in a diamondoid network. Figure (**a**) shows a repeating unit of the diamondoid network, i.e., an adamantane unit consisting of 10 vertices, while (**b**) depicts two vertex-shared adamantane units. Figure (**c**,**d**) show the diamondoid network looking along the crystallographic *b* axis, with (**c**) also elucidating connections among these clusters.

From a topological perspective, we may consider Ca′ (Ca2) as a tetrahedral 4-connecting node (type C node, presented as cyan in Figure 6) that is linked to a pair of neighboring equivalents in the crystallographic *b* direction (within the rod-shaped chain, Figure 6c,d) and a pair of [Cd_2_Ca] (type A node presented as orange in Figure 6). The four angles around the Ca′ (Ca2) are θ1 = 136°, θ2 = θ3 = 99°, and θ4 = 152° (Figure 6b). Four type A nodes are arranged in a head-to-head fashion to generate a small A_4_ motif. This A_4_ motif also functions as a tetrahedral node and extends to four type C nodes, and ultimately gives rise to a diamond-type 3D network.

It should be noted that the MOF **1** structure reported herein is drastically different from, and as a matter of fact more complicated than, several BTB-based MOFs of Cd, such as 2D MOF of [Cd_3_(BTB)_2_(DEF)_4_]·2(DEF)_0.5_ [62,63,64], 3D MOF of [Et_2_NH_2_]_2_[Cd_5_(BTB)_4_(DEF)_2_]∙4.75DEF [65], and 3D MOF of [NEt_4_]_2_[Cd_5_(BTB)_4_(H_2_O)_4_] [64]. We are not aware of any BTB-based MOFs of Ca reported to date.

## 3. Synthesis and Material Characterization

### 3.1. General

Cd(NO_3_)_2_·4H_2_O (≥ 99.99%, Macklin), benzene-1,3,5-tribenzoic acid (H_3_BTB, > 98.0%, TCI), *N*,*N*′-diethylformamide (DEF, > 99.0%, TCI), CaO (98.0%, Macklin), and HNO_3_ (65.0−68.0%, Enox) were obtained from commercial sources and used as received. Fourier-transform infrared (FT-IR) spectrum was measured on a Varian 1000 FT-IR spectrometer (Varian, Inc., Palo Alto, CA, USA) as KBr disks (400−4000 cm^−1^). Elemental analyses for C, H, and N were conducted on a Carlo-Erba CHNO-S microanalyzer (Carlo Erba, Waltham, MA, USA), with the sample first immersed in CHCl_3_ to replace the encapsulated species with volatile CHCl_3_ and air-dried before analysis. The thermogravimetric analysis (TGA) was performed on a TA instrument Q500 (TA instruments, New Castle, DE, USA) from room temperature to 800 °C at a heating rate of 10 °C min^−1^ under an N_2_ gas flow in an Al_2_O_3_ pan. The powder X-ray diffraction (PXRD) pattern was recorded on a Bruker D8 GADDS (General Area Detector Diffraction System) micro-diffractometer (Bruker AXS GmbH, Karlsruhe, Germany) equipped with a VANTEC-2000 area detector (Bruker AXS GmbH, Karlsruhe, Germany) using the Φ rotation method. Nitrogen adsorption isotherms were recorded using a BELSORP-max (MicrotracBEL Corp., Osaka, Japan). The encapsulated solvent in the sample was exchanged with CHCl_3_ twice and air-dried in the fumehood, before being transferred to the instrument for activation at room temperature under a vacuum of 10^−2^ kPa. The evacuated sample tube was weighed again after 36 h and the sample mass was determined by subtracting the original mass. The nitrogen isotherms were measured using a liquid nitrogen bath (77 K).

### 3.2. Synthesis of [Cd_6_Ca_4_(BTB)_6_(HCOO)_2_(DEF)_2_(H_2_O)_12_]∙DEF∙xSol. (MOF **1**)

Cd(NO_3_)_2_·4H_2_O (31 mg, 0.1 mmol), CaO (2.8 mg, 0.05 mmol) and H_3_BTB (44 mg, 0.1 mmol) were added to a mixture of DEF/H_2_O/HNO_3_ (6.5 mL/1.0 mL/50 μL) in a sealed pressure tube (25 mL). The reaction was heated to 85 °C in an oven for 72 h and cooled to room temperature over 48 h to give colorless block crystals of [Cd_6_Ca_4_(BTB)_6_(HCOO)_2_(DEF)_2_(H_2_O)_12_]∙DEF∙*x*Sol. (**1**). Yield 44 mg, 87% based on Ca. Anal. Calcd (%) for [Cd_6_Ca_4_(BTB)_6_(HCOO)_2_(DEF)_2_(H_2_O)_12_]∙DEF∙2CHCl_3_∙8H_2_O: C 48.97, H 3.79, N 0.95; found: C 48.71, H 4.31, N 0.96. IR (KBr disk, cm^−1^): 3416 (br), 3065 (m), 2975 (w), 2930 (m), 1937 (w), 1812 (w), 1652(s), 1610 (sh), 1582(s), 1536 (s), 1397 (vs), 1303 (m), 1265 (m), 1210 (m), 1180 (m), 1146 (w), 1105 (m), 893 (w), 855 (m), 812 (m), 780 (s), 704 (m), 670 (w), 649 (w), 540 (w), 471(w).

### 3.3. X-Ray Crystallography for MOF **1**

The single-crystal structure of MOF **1** was analyzed on a Bruker D8 Quest CCD X-ray diffractometer with graphite monochromated Mo Kα (λ = 0.71073 Å) radiation. Refinement and reduction of the collected data were achieved using the Bruker SAINT program and applied to all complexes with absorption correction (multi-scan) [75]. The crystal structure was solved by direct methods and refined on *F*^2^ by full-matrix least-squares techniques with the SHELXTL-2013 program [76]. In MOF **1**, the two coordinated waters (O25 and O26) display positional disorder with relative ratios of 0.54/0.46 refined for the two components. The occupancy factors for the atoms of the dissociated DEF solvate were fixed at 0.5 to obtain reasonable thermal factors. The hydrogen atoms on the water molecules (O21, O23, and O24) were located from the difference Fourier map, while those on the water molecule (O22) were generated by considering their possible hydrogen-bonding interactions with atoms nearby. The O–H distances and thermal parameters were subsequently constrained to O–H = 0.83 Å and *U*_iso_(H) = 1.2*U*_eq_(O). The hydrogen atoms on the disordered water solvates (O25, O26/O25a, O26a) were not located. The structure adopted non-merohedral twinning about the (−1 −1 0) and the twin law [0 −1 0 −1 0 0 0 0 −1] was included during the refinement. A large amount of spatially delocalized electron density in the lattice was also found (3247 electrons in 20,271 Å^3^ solvent-accessible volume), but acceptable refinement could not be obtained for this electron density. The solvent contribution was then modeled using SQUEEZE in the Platon program suite [77]. Crystallographic data were deposited with the Cambridge Crystallographic Data Center as supplementary publication number CCDC 1990807. These data can be obtained free of charge from the Cambridge Crystallographic Data Centre via www.ccdc.cam.ac.uk/data_request/cif. A summary of the key crystallographic data is listed in Table 1, and selected bond lengths and angles are depicted in Supplementary Table S1.

## 4. Conclusions

The isolation of MOF **1** exemplified the usefulness of s-block metal ions for the MOF assembly. The three independent Ca^2+^ ions in MOF **1** exhibit coordination numbers of either six (Ca1 and Ca3) or eight (Ca2), with the latter seldom achieved for a typical first or second-row d-block metal complex. The structure of MOF **1** is complex and dramatically different from MOFs exclusively based on Cd-BTB. Cd^2+^ and Ca^2+^, although similar in size, are quite different in atomic mass, which made them easy to distinguish during the X-ray crystal structure analysis. MOF **1** was insufficiently stable for complete removal of solvate from the large pore, which prevented us from obtaining meaningful porosity data via gas adsorption studies. Nevertheless, we believe that careful selection of s-block metals to mix with d-block metal ions (not limited to two) could be a powerful strategy for preparing MOFs with interesting topologies and unique properties.

## Figures and Tables

**Table 1:** Crystallographic data and refinement parameters for MOF **1**.

Parameter	Value
Molecular formula	C_179_H_141_Ca_4_Cd_6_N_3_O_55_
Formula weight	4048.66
Crystal system	Tetragonal
Space group	*I*4_1_/*a*
*a* (Å)	30.7009 (10)
*b* (Å)	30.7009 (10)
*c* (Å)	54.251 (4)
α (°)	90
*β* (°)	90
γ (°)	90
*V* (Å^3^)	51,134 (5)
Z	8
*ρ*_calc_ (g cm^−3^)	1.052
*F*(000)	16,352
*µ* (mm^−1^)	0.629
Total reflections	438,339
Unique reflections	31,735
Observed reflections	28,071
*R*_int_	0.1169
Variables	1154
*R*_1_ *^a^*	0.0633
*wR*_2_ *^b^*	0.1615
GOF *^c^*	1.087

*^a^ R_1_* = Σ||*F*_o_|−|*F*_c_||/Σ|*F*_o_|, *^b^ wR*_2_ = {Σ[*w*(*F*_o_^2^ − *F*_c_^2^)^2^]/Σ[*w*(*F*_o_^2^)^2^]}^1/2^, *^c^* GOF = {Σ[*w*(*F*_o_^2^ − *F*_c_^2^)^2^]/(*n* − *p*)}^1/2^, where *n* is the number of reflections and *p* is total number of parameters refined.

## Supplementary Materials

Figure S1: The TGA curve of MOF **1** showing that the framework is stable up to ca. 100 °C, followed by continuous weight loss and framework decomposition until ca. 650 °C; Figure S2: The N_2_ (77 K) sorption isotherms of MOF **1** with the black squares and red circles representing adsorption and desorption. P_0_ is the saturated vapor pressure of the adsorbates at the measurement temperatures; Table S1: Selected bond distances in MOF **1** involving the Cd and Ca centers.
