# Supplementary material for: A Heterometallic Three-Dimensional Metal−Organic Framework Bearing an Unprecedented One-Dimensional Branched-Chain Secondary Building Unit
Source: Molecules. 2020 May 7;25(9):2190. doi: 10.3390/molecules25092190 (PMC7248776; doi:10.3390/molecules25092190)
Supplement: Supplementary file 1 [file molecules-25-02190-s001.pdf]

Supporting Information for

A Heterometallic Three-Dimensional Metal–Organic Framework Bearing an Unprecedented One-Dimensional Branched-Chain Secondary Building Unit

Jing Chen[1,#], Meng-Yao Chao[1,#], Yan Liu[1], Bo-Wei Xu[1], Wen-Hua Zhang[1,*] and David J. Young[2]

[1] College of Chemistry, Chemical Engineering and Materials Science, Soochow University, Suzhou 215123, China

[2] College of Engineering, Information Technology & Environment, Charles Darwin University, Darwin, Northern Territory 0909, Australia

* Correspondence: chenjing120905003@163.com (J.C.); chaomy@mail.sustech.edu.cn (M.-Y.C.); liuyanllllll@163.com (Y.L.); huhulalaxu@163.com (B.-W.X.); whzhang@suda.edu.cn (W.-H.Z.); david.young@cdu.edu.au (D.J.Y.)

\# These authors contributed equally to this work.

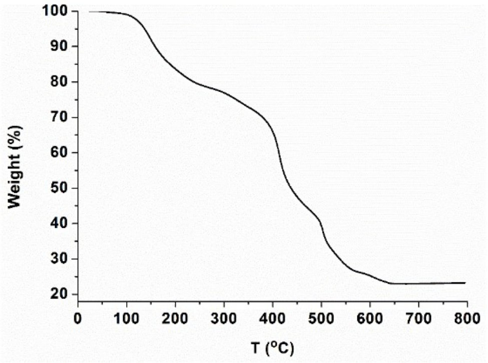

Figure S1. The TGA curve of MOF **1** showing that the framework is stable up to ca. 100 °C, followed by continuous weight loss and framework decomposition until ca. 650 °C.

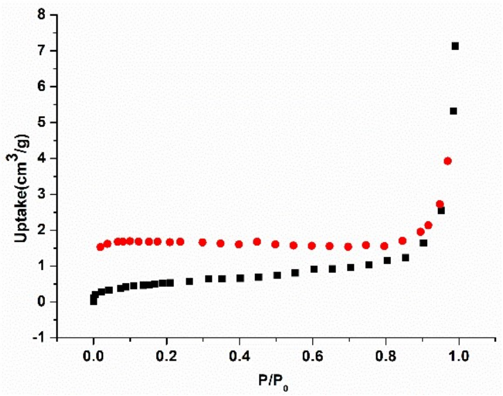

Figure S2. The N_2 (77 K) sorption isotherms of MOF **1** with the black squares and red circles representing adsorption and desorption. P_0 is the saturated vapor pressure of the adsorbates at the measurement temperatures.

Table S1. Selected bond distances in MOF **1** involving the Cd and Ca centers.

Cd(1)-O(15)#1	2.236(4)	Cd(1)-O(9)#2	2.251(4)
Cd(1)-O(18)#3	2.323(4)	Cd(1)-O(21)	2.334(5)
Cd(1)-O(22)	2.361(5)	Cd(1)-O(17)#3	2.495(4)
Cd(1)-Ca(1)	3.8452(11)	Cd(2)-O(3)#4	2.230(4)
Cd(2)-O(19)	2.286(4)	Cd(2)-O(24)	2.320(6)
Cd(2)-O(1)	2.323(5)	Cd(2)-O(6)#5	2.381(4)
Cd(2)-O(5)#5	2.400(4)	Cd(2)-O(2)	2.532(4)
Cd(2)-Ca(1)	3.5774(11)	Cd(3)-O(11)#6	2.199(4)
Cd(3)-O(20)	2.244(4)	Cd(3)-O(7)	2.249(4)
Cd(3)-O(13)	2.322(4)	Cd(3)-O(14)	2.394(4)
Cd(3)-O(27)	2.585(13)	Ca(1)-O(10)#2	2.271(4)
Cd(3)-Ca(3)	3.5471(4)	Ca(1)-O(2)	2.281(4)
Ca(1)-O(4)#4	2.272(4)	Ca(1)-O(17)#3	2.340(4)
Ca(1)-O(6)#5	2.329(4)	Ca(2)-O(26)	2.381(8)
Ca(1)-O(23)	2.343(5)	Ca(2)-O(26)#7	2.381(7)
Ca(2)-O(25)	2.437(9)	Ca(2)-O(25)#7	2.437(9)
Ca(2)-O(19)#7	2.476(4)	Ca(2)-O(19)	2.476(4)
Ca(2)-O(20)	2.513(4)	Ca(2)-O(20)#7	2.513(4)
Ca(3)-O(8)	2.253(4)	Ca(3)-O(8)#3	2.253(4)
Ca(3)-O(13)#3	2.309(4)	Ca(3)-O(13)	2.309(4)
Ca(3)-O(12)#8	2.312(3)	Ca(3)-O(12)#6	2.312(3)
O(15)#1-Cd(1)-O(9)#2	97.03(17)	O(15)#1-Cd(1)-O(18)#3	95.12(17)
O(9)#2-Cd(1)-O(18)#3	166.81(16)	O(15)#1-Cd(1)-O(21)	91.06(19)
O(9)#2-Cd(1)-O(21)	94.8(2)	O(18)#3-Cd(1)-O(21)	90.1(2)
O(15)#1-Cd(1)-O(22)	174.56(17)	O(9)#2-Cd(1)-O(22)	83.51(17)
O(18)#3-Cd(1)-O(22)	84.88(16)	O(21)-Cd(1)-O(22)	83.50(19)
O(15)#1-Cd(1)-O(17)#3	95.20(16)	O(9)#2-Cd(1)-O(17)#3	119.67(15)
O(18)#3-Cd(1)-O(17)#3	53.81(13)	O(21)-Cd(1)-O(17)#3	143.7(2)
O(22)-Cd(1)-O(17)#3	89.20(15)	O(3)#4-Cd(2)-O(24)	169.8(2)
O(3)#4-Cd(2)-O(19)	83.26(19)	O(19)-Cd(2)-O(24)	86.7(2)
O(3)#4-Cd(2)-O(1)	92.0(2)	O(19)-Cd(2)-O(1)	90.24(18)
O(24)-Cd(2)-O(1)	85.9(2)	O(3)#4-Cd(2)-O(6)#5	100.07(17)
O(19)-Cd(2)-O(6)#5	144.08(16)	O(24)-Cd(2)-O(6)#5	89.3(2)
O(1)-Cd(2)-O(6)#5	125.07(15)	O(3)#4-Cd(2)-O(5)#5	101.38(18)
O(19)-Cd(2)-O(5)#5	89.64(16)	O(24)-Cd(2)-O(5)#5	80.6(2)
O(1)-Cd(2)-O(5)#5	166.49(18)	O(6)#5-Cd(2)-O(5)#5	54.53(13)
O(3)#4-Cd(2)-O(2)	88.79(17)	O(19)-Cd(2)-O(2)	142.69(16)
O(24)-Cd(2)-O(2)	98.0(2)	O(1)-Cd(2)-O(2)	53.57(15)
O(6)#5-Cd(2)-O(2)	73.20(13)	O(5)#5-Cd(2)-O(2)	127.67(14)
O(11)#6-Cd(3)-O(20)	90.86(15)	O(11)#6-Cd(3)-O(7)	94.22(16)
O(20)-Cd(3)-O(7)	101.18(17)	O(11)#6-Cd(3)-O(13)	116.44(15)
O(20)-Cd(3)-O(13)	138.89(14)	O(7)-Cd(3)-O(13)	106.37(17)
O(11)#6-Cd(3)-O(14)	161.81(16)	O(20)-Cd(3)-O(14)	89.49(14)

O(7)-Cd(3)-O(14)	103.56(16)	O(13)-Cd(3)-O(14)	54.93(14)
O(11)#6-Cd(3)-O(27)	81.6(4)	O(20)-Cd(3)-O(27)	77.0(3)
O(7)-Cd(3)-O(27)	175.3(4)	O(13)-Cd(3)-O(27)	77.5(3)
O(14)-Cd(3)-O(27)	80.8(4)	O(10)#2-Ca(1)-O(4)#4	173.52(19)
O(10)#2-Ca(1)-O(2)	101.04(18)	O(4)#4-Ca(1)-O(2)	85.42(18)
O(10)#2-Ca(1)-O(6)#5	97.58(16)	O(4)#4-Ca(1)-O(6)#5	84.13(16)
O(2)-Ca(1)-O(6)#5	78.97(15)	O(10)#2-Ca(1)-O(17)#3	81.01(15)
O(4)#4-Ca(1)-O(17)#3	97.85(16)	O(2)-Ca(1)-O(17)#3	96.16(15)
O(6)#5-Ca(1)-O(17)#3	174.61(16)	O(10)#2-Ca(1)-O(23)	88.99(19)
O(4)#4-Ca(1)-O(23)	84.59(18)	O(2)-Ca(1)-O(23)	169.73(18)
O(6)#5-Ca(1)-O(23)	97.66(17)	O(17)#3-Ca(1)-O(23)	87.54(17)
O(26)-Ca(2)-O(26)#7	138.3(4)	O(26)-Ca(2)-O(25)	51.3(3)
O(26)#7-Ca(2)-O(25)	159.9(3)	O(26)-Ca(2)-O(25)#7	159.9(3)
O(26)#7-Ca(2)-O(25)#7	51.3(3)	O(25)-Ca(2)-O(25)#7	127.6(5)
O(26)-Ca(2)-O(19)#7	126.4(2)	O(26)#7-Ca(2)-O(19)#7	70.7(2)
O(25)-Ca(2)-O(19)#7	89.8(3)	O(25)#7-Ca(2)-O(19)#7	71.3(2)
O(26)-Ca(2)-O(19)	70.7(2)	O(26)#7-Ca(2)-O(19)	126.4(2)
O(25)-Ca(2)-O(19)	71.3(2)	O(25)#7-Ca(2)-O(19)	89.8(3)
O(19)#7-Ca(2)-O(19)	136.9(3)	O(26)-Ca(2)-O(20)	89.0(2)
O(26)#7-Ca(2)-O(20)	80.05(19)	O(25)-Ca(2)-O(20)	119.9(2)
O(25)#7-Ca(2)-O(20)	74.8(2)	O(19)#7-Ca(2)-O(20)	144.43(14)
O(19)-Ca(2)-O(20)	51.92(14)	O(26)-Ca(2)-O(20)#7	80.05(19)
O(26)#7-Ca(2)-O(20)#7	89.0(2)	O(25)-Ca(2)-O(20)#7	74.8(2)
O(25)#7-Ca(2)-O(20)#7	119.9(2)	O(19)#7-Ca(2)-O(20)#7	51.92(14)
O(19)-Ca(2)-O(20)#7	144.43(14)	O(20)-Ca(2)-O(20)#7	149.0(2)
O(8)-Ca(3)-O(8)#3	180.00(19)	O(8)-Ca(3)-O(13)#3	99.01(16)
O(8)#3-Ca(3)-O(13)#3	80.99(16)	O(8)-Ca(3)-O(13)	80.99(16)
O(8)#3-Ca(3)-O(13)	99.01(16)	O(13)#3-Ca(3)-O(13)	180.0
O(8)-Ca(3)-O(12)#8	95.32(15)	O(8)#3-Ca(3)-O(12)#8	84.69(15)
O(13)#3-Ca(3)-O(12)#8	86.69(15)	O(13)-Ca(3)-O(12)#8	93.32(15)
O(8)-Ca(3)-O(12)#6	84.68(15)	O(8)#3-Ca(3)-O(12)#6	95.31(15)
O(13)#3-Ca(3)-O(12)#6	93.31(15)	O(13)-Ca(3)-O(12)#6	86.68(15)
O(12)#8-Ca(3)-O(12)#6	180.00(11)	O(8)-Ca(3)-Cd(3)	53.60(12)
O(8)#3-Ca(3)-Cd(3)	126.40(12)	O(13)#3-Ca(3)-Cd(3)	139.84(10)
O(13)-Ca(3)-Cd(3)	40.15(10)	O(12)#8-Ca(3)-Cd(3)	120.90(10)
O(12)#6-Ca(3)-Cd(3)	59.10(10)	O(8)-Ca(3)-Cd(3)#3	126.40(12)
O(8)#3-Ca(3)-Cd(3)#3	53.60(12)	O(13)#3-Ca(3)-Cd(3)#3	40.16(10)
O(13)-Ca(3)-Cd(3)#3	139.85(10)	O(12)#8-Ca(3)-Cd(3)#3	59.10(10)
O(12)#6-Ca(3)-Cd(3)#3	120.90(10)	Cd(3)-Ca(3)-Cd(3)#3	180.0

Symmetry Codes: #1 $-y + 3/4$, $x + 3/4$, $-z + 7/4$; #2 x, $y - 1/2$, $-z + 2$; #3 $-x$, $-y + 1$, $-z + 2$; #4 $-y + 3/4$, $x + 1/4$, $z + 1/4$; #5 $-y + 3/4$, $x-1/4$, $-z + 7/4$; #6 $y - 1/4$, $-x + 3/4$, $z - 1/4$; #7 $-x$, $-y + 1/2$, z; #8 $-y + 1/4$, $x + 1/4$, $-z + 9/4$.
